# In Situ Construction of Hollow Coral‐Like Porous S‐Doped g‐C_3_N_4_/ZnIn_2_S_4_ S‐Scheme Heterojunction for Efficient Photocatalytic Hydrogen Evolution

**DOI:** 10.1002/advs.202403771

**Published:** 2024-07-03

**Authors:** Tianyu Wang, Xuanlin Pan, Minyi He, Lei Kang, Wangjing Ma

**Affiliations:** ^1^ Technical Institute of Physics and Chemistry Chinese Academy of Sciences Zhongguancun East Road, Haidian District Beijing 100190 China; ^2^ University of Chinese Academy of Sciences Zhongguancun East Road, Haidian District Beijing 100049 China

**Keywords:** flexible C─S─C bond, hollow coral‐like porous structure, synergistic effect

## Abstract

The rational design of visible‐light‐responsive catalysts is crucial for converting solar energy into hydrogen energy to promote sustainable energy development. In this work, a C─S─C bond is introduced into g‐C_3_N_4_ (CN) through S doping. With the help of the flexible C─S─C bond under specific stimuli, a hollow coral‐like porous structure of S‐doped g‐C_3_N_4_ (S‐CN) is synthesized for the first time. And an S‐doped g‐C_3_N_4_/ZnIn_2_S_4_ (S‐CN/ZIS) heterojunction catalyst is in situ synthesized based on S‐CN. S_0.5_‐CN/ZIS exhibits excellent photocatalytic hydrogen evolution (PHE) efficiency (19.25 mmol g^−1^ h^−1^), which is 2.7 times higher than that of the g‐C_3_N_4_/ZnIn_2_S_4_ (CN/ZIS) catalyst (8.46 mmol g^−1^ h^−1^), with a high surface quantum efficiency (AQE) of 34.43% at 420 nm. Experiments and theoretical calculations demonstrate that the excellent photocatalytic performance is attributed to the larger specific surface area and porosity, enhanced interfacial electric field (IEF) effect, and appropriate hydrogen adsorption Gibbs free energy (ΔG_H*_). The synergistic effect of S doping and S‐scheme heterojunction contributes to the above advancement. This study provides new insights and theoretical basis for the design of CN‐based photocatalysts.

## Graphical abstract

S‐CN/ZIS has a hollow coral‐like porous structure. ZIS uniformly grows on the surface of S‐CN to construct an S‐scheme heterojunction. Compared to CN/ZIS, S‐CN/ZIS exhibits a stronger interface electric field effect, larger specific surface area, and more suitable hydrogen adsorption and desorption energy. Therefore, S‐CN/ZIS can achieve efficient photocatalytic hydrogen evolution.

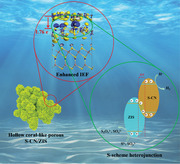

## 1 Introduction

To address the increasingly severe energy crisis and environmental pollution today, developing new sustainable energy sources is extremely necessary. Hydrogen energy is considered a potential sustainable energy source due to its clean, efficiency, safety, and high energy density.^[^ ^1^ ^]^ Among the various methods of obtaining hydrogen energy, photocatalytic water splitting for hydrogen production is an ideal approach.^[^ ^2^ ^]^ Under light irradiation, semiconductor photocatalysts can continuously split water into H_2_, thus obtaining hydrogen energy sustainably without pollution.^[^ ^3^ ^]^ Among many photocatalysts for hydrogen production, CN has attracted attention due to its good stability, narrow band gap, and sufficient reduction capability.^[^ ^4^ ^]^ However, pure CN is a bulk structure consisting of stacked nanosheets, which is lack of sufficient reaction sites.^[^ ^5^ ^]^ Therefore, a rational design of CN morphology is necessary to enhance its PHE activity.^[^ ^6^ ^]^ In recent years, hollow nanostructures have been widely used in fields such as microwave absorption,^[^ ^7^ ^]^ catalysis,^[^ ^8^ ^]^ and drug delivery^[^ ^9^ ^]^ due to their fascinating characteristics. For photocatalysis, the hollow structure allows light to continuously reflect and scatter within the cavity, enhancing the absorption of incident light.^[^ ^10^ ^]^ The thin shell can reduce the migration distance of charge carriers and suppress charge recombination.^[^ ^11^ ^]^ The large specific surface area also provides more active sites for catalytic reactions.^[^ ^12^ ^]^ Therefore, the hollow structure could be a great alternative to improve the PHE efficiency of CN. Although hollow tubular,^[^ ^13^ ^]^ hollow sphere,^[^ ^14^ ^]^ and honeycomb‐like^[^ ^6^, ^15^ ^]^ structures of CN have been proven that they are effective to improve the PHE, their preparation methods are complex and contribution to photocatalytic activity is still limited. Recently, the C─S─C bond has been shown to induce molecular conformational distortion, leading to folding of crystal structures, and thus morphology could be improved.^[^ ^16^ ^]^ However, few research has been reported on morphology modification of carbon‐based materials like CN using C─S─C bonds.

Besides, constructing heterojunction is considered an effective method for enhancing photocatalytic activity of CN.^[^ ^17^ ^]^ As is well known, it is difficult for single‐component photocatalysts to achieve a high hydrogen production rate due to the contradiction between light absorption and charge carrier separation. In contrast, heterojunction photocatalysts have strong light absorption from different components, which can also promote charge transfer and separation through the interface electric field effect, thereby improving photocatalytic performance.^[^ ^18^ ^]^ Based on the partial overlap of the energy bands in CN and ZIS, Tan et al. prepared a ZIS/CN heterojunction photocatalyst for efficient visible‐light‐catalyzed hydrogen production.^[^ ^19^ ^]^ The formation of a Z‐scheme heterojunction promotes synergistic interaction between CN and ZIS, resulting in an excellent PHE efficiency of ZIS/CN heterojunction photocatalysts. 2D/2D structure provides a larger contact area to promote the separation and migration of charge carriers. However, this structure has limited light utilization efficiency and specific surface area. Meanwhile, the small difference in the work function weakens the IEF, which makes it hard to drive the migration of charge carriers. Fortunately, relevant studies have shown that S doping can reduce the work function of CN to increase IEF intensity between CN and ZIS,^[^ ^20^ ^]^ thereby improving the separation and migration efficiency of photogenerated charge carriers.

In this study, S‐CN with a hollow coral‐like porous structure was successfully prepared by rapid recrystallization of urea and TAA in a mixed solution, together with calcination. Subsequently, ZIS was grown in situ on the S‐CN framework to prepare the S‐CN/ZIS heterojunction. The surface folds and internal cavities of S‐CN provide a large specific surface area, more exposed active sites, and high light utilization efficiency. Constructing heterojunctions also accelerates the separation and migration of photogenerated charge carriers. Furthermore, the synergistic effect of S‐doping and S‐scheme heterojunction perfectly remain the advantages of S‐doping to S‐CN/ZIS, and further promotes the charge transfer of S‐scheme heterojunction. Therefore, the hydrogen production rate of the S_0.5_‐CN/ZIS under visible light irradiation reaches up to 19.25 mmol g^−1^ h^−1^, which is 2.27 times higher than that of the CN/ZIS catalyst. In addition, the AQE at a wavelength of 420 nm reaches 34.43%, which is superior to similar catalysts in previous studies. Moreover, a thorough investigation was conducted on the microstructure of the composite material, enhanced IEF effect, H adsorption Gibbs free energy, and charge transfer mechanism of the S‐scheme heterojunction. This study provides a new perspective and theoretical basis for the design of efficient CN‐based photocatalysts.

## 2 Experimental Section

### 2.1 Preparation of Catalysts

#### 2.1.1 Synthesis of S Doped g‐C_3_N_4_ and g‐C_3_N_4_

Urea (15 g) were dissolved in 20 mL of deionized water and stirred vigorously. The solution was heated at 50 °C for 30 min and different mass of thioacetamide (TAA) were added. The solution was quickly dried under microwave to form a transparent precursor. The precursor was transformed to a semi‐closed crucible and heated at 300 °C for 1 h. Then, the temperature was increased to 500 °C at a rate of 5 °C min^−1^ and maintained for 2 h. After cooling to room temperature, the product was washed several times with deionized water and ethanol, then dried in an oven at 60 °C overnight. The final products (S_x_‐CN) were obtained after drying, where x represents different S doping molar ratios (1%, 0.75%, 0.5%, and 0.25%). For comparison, pure CN was prepared under the same conditions without adding TAA.

#### 2.1.2 Synthesis of S Doped g‐C_3_N_4_/ZnIn_2_S_4_, g‐C_3_N_4_/ZnIn_2_S_4_, and ZnIn_2_S_4_

The S‐CN/ZIS composites were prepared using a hydrothermal method. S‐CN was dispersed in 40 mL of deionized water and sonicated for 30 min. Subsequently, ZnCl_2_ (0.8 mmol), InCl_3_·4H_2_O (1.6 mmol), and TAA (6.8 mmol) were sequentially added and stirred for 3 h. The mixture was transferred to a 100 mL Teflon‐lined stainless steel autoclave and heated at 160 °C for 1 h. After air cooling, the product was obtained through centrifugation, then washed with deionized water and ethanol, and subsequently dried in air at 60 °C overnight. For comparison, ZIS was synthesized under the same conditions without adding S‐CN, while CN/ZIS was synthesized using pure CN.

### 2.2 Characterization Information

Characterization information can be found in Text S2 (Supporting Information).

## 3 Results and Discussion

### 3.1 Structural Characterization and Surface Properties

**Figure 1:**
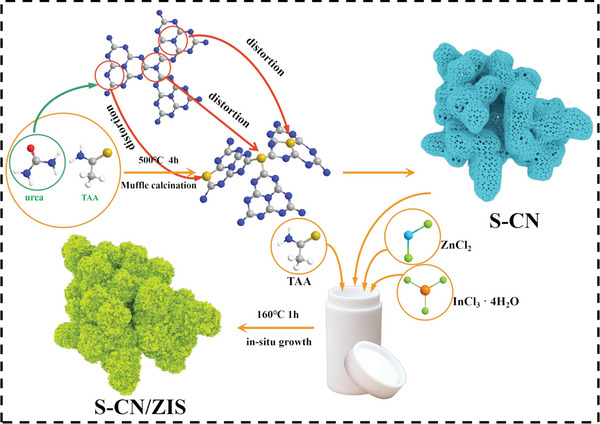
a) Schematic diagram of the synthetic route of S‐CN/ZIS.

**Figure 2:**
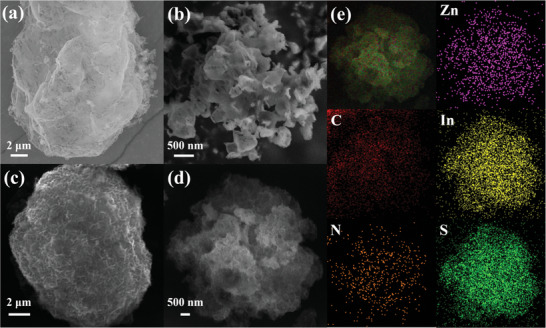
SEM of a) CN, b) S_0.5_‐CN, c) CN/ZIS, and d) S_0.5_‐CN/ZIS. e) SEM‐EDS elemental maps of S_0.5_‐CN/ZIS composite catalyst.

As shown in **Figure** **1**, the preparation of hollow coral‐like porous S‐CN/ZIS includes two steps. First, the hollow coral‐like porous S‐CN framework was synthesized through rapid recrystallization of urea and TAA in a mixed solution, together with calcination. As shown in **Figures** **2**a and S1a (Supporting Information), pure CN has an irregular block structure, and the stacking of nanosheets is not conducive to the exposure of active sites. With the introduction of S atoms into the lattice, S_0.5_‐CN presents a coral‐like porous structure (Figure 2b). The increase in surface folds and porosity is beneficial for the exposure of active sites and the adsorption of water molecules (Figure S1b, Supporting Information). To investigate the effect of temperature on the flexibility of the C─S─C bond, S_0.5_‐CN was synthesized at 450 and 550 °C, respectively. As shown in Figure S2 (Supporting Information), S_0.5_‐CN (450 °C) still exhibits a block‐like structure, but the surface has a tendency to show folding deformation, gradually leading to the appearance of voids. In contrast, S_0.5_‐CN (550 °C) completely peels off into flakes (Figure S3, Supporting Information), indicating that the flexibility of C─S─C bond could be influenced by temperature. ZIS presents a nano‐flower structure formed by stacked nano‐sheets (Figure S4, Supporting Information). Subsequently, ZIS nanosheets were grown in situ on the surface of S_0.5_‐CN through a hydrothermal method to prepare the S_0.5_‐CN/ZIS heterojunction (Figure 2d; Figure S1d, Supporting Information). As a comparison, we synthesized the CN/ZIS heterojunction, where ZIS uniformly grows on the surface of the bulk material (Figure 2c; Figure S1c, Supporting Information). It is evident that the surface folds and voids of S_0.5_‐CN are more conducive to the growth of ZIS and their contact, promoting charge transfer at the interface. As shown in Figure 2e, the elements C, N, Zn, In, and S are evenly distributed on the surface of S_0.5_‐CN/ZIS. However, the density of Zn, In, and S is higher than that of C and N, demonstrating that the S_0.5_‐CN framework is internal, while ZIS covered the surface of S_0.5_‐CN.

**Figure 3:**
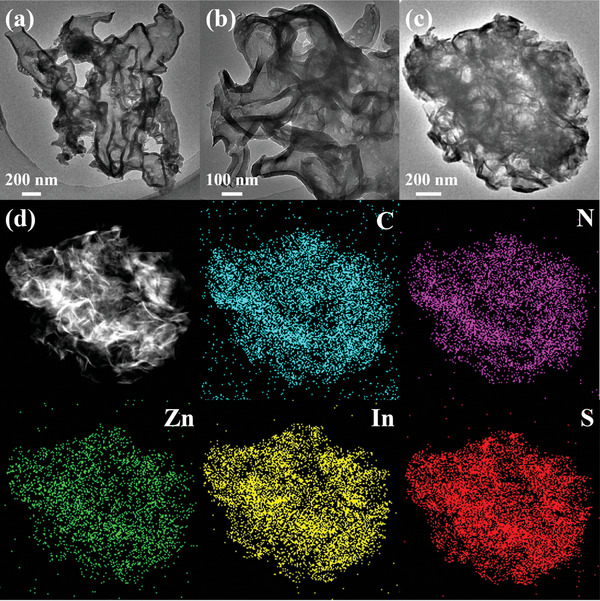
TEM of a) CN, b) S_0.5_‐CN, and c) S_0.5_‐CN/ZIS. d) TEM‐EDS elemental maps of S_0.5_‐CN/ZIS composite catalyst.

**Figure 4:**
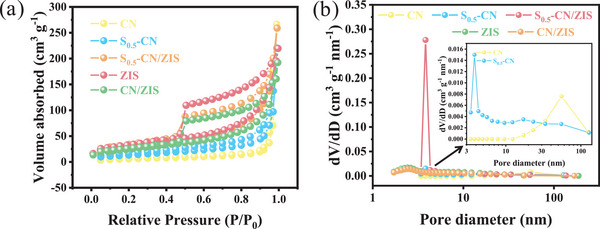
a) N_2_ adsorption–desorption isotherms and b) pore size distributions of CN, S_0.5_‐CN, ZIS, CN/ZIS, and S_0.5_‐CN/ZIS composites.

The internal structure of the sample can be observed more clearly by transmission electron microscopy (TEM). As shown in **Figure** **3**a, cavities inside S_0.5_‐CN could be observed obviously, and the color of the outer surface darkens due to surface folding. Many small pores can be clearly observed on the surface of S_0.5_‐CN (Figure 3b), enabling light to enter the cavities through the pores and undergo multiple reflections, which improves light utilization. Figure 3c shows the TEM image of S_0.5_‐CN/ZIS which retains the framework of S_0.5_‐CN, while ZIS was grown uniformly on the framework. High‐resolution transmission electron microscopy (HRTEM) images (Figure S5, Supporting Information) reveal a lattice spacing of 0.32 nm for ZIS, corresponding to the (102) crystal plane of ZIS.^[^ ^19^ ^]^ The image illustrates the clear interface between ZIS nanosheets and S_0.5_‐CN, depicting the successful coupling of ZIS and S_0.5_‐CN. As shown in Figure 3d, C, N, Zn, In, and S are uniformly distributed with similar densities, contrasting sharply with the images observed by the SEM‐EDS elemental distribution map, proving that ZIS is loaded on the surface of the S_0.5_‐CN framework. It can also be observed in the TEM‐EDS line scan. As shown in Figure S6 (Supporting Information), the distribution of Zn, In, and S elements in the outer layer of the sample is higher than that of C and N, while the inner layer of the sample has a more homogeneous content of each element (Figure S7, Supporting Information). The nitrogen adsorption–desorption curve can more clearly show the changes in specific surface area and porosity. As shown in **Figure** **4**a, except for CN, all other samples belong to Type IV isotherm, indicating the presence of mesopores in the samples. Moreover, samples containing S_0.5_‐CN have a larger specific surface area, which means more active sites could be exposed. As Figure 4b, the pore size of S_0.5_‐CN is significantly smaller than that of CN, while the pore size of S_0.5_‐CN/ZIS is closed to that of S_0.5_‐CN. More importantly, S_0.5_‐CN/ZIS has the largest pore volume, indicating that it has the largest effective adsorption volume, which is significantly beneficial for the adsorption and activation of water molecules (Table S1, Supporting Information).

**Figure 5:**
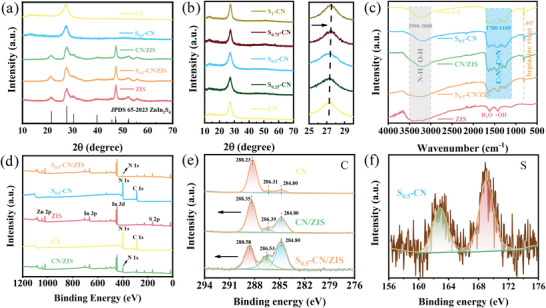
XRD patterns of a) ZIS, CN, S_0.5_‐CN, CN/ZIS, S_0.5_‐CN/ZIS, and b) S_x_‐CN, c) FTIR of ZIS, CN, S_0.5_‐CN, CN/ZIS, and S_0.5_‐CN/ZIS, d) XPS survey spectra of ZIS, CN, S_0.5_‐CN, CN/ZIS, and S_0.5_‐CN/ZIS, and High‐resolution XPS spectra of e) C 1S, (f) S 2p.

The crystal structures of CN, S‐CN, ZIS, CN/ZIS, and S‐CN/ZIS samples were analyzed using X‐ray diffraction (XRD). As shown in **Figure** **5**a, the peaks at 21.3°, 27.4°, and 47.1° correspond to the (006), (102), and (110) crystal planes of hexagonal ZIS (JCPDS 65–2023, a = b = 3.85 Å, c = 24.68 Å).^[^ ^21^ ^]^ As shown in Figure 5b, the shape and position of the S‐CN diffraction peaks are similar to those of CN, but the main diffraction peak (002) is shifted to a higher angle with increasing S doping, indicating that S doping leads to lattice distortion. This implies the presence of lattice defects in S‐CN, which favors the separation of photogenerated electron‐hole pairs. Due to the overlap of the (002) crystal plane in CN with the (102) crystal plane of ZIS, the peak intensity at 27.4° in the composite material is stronger than in ZIS. The characteristic functional groups of the photocatalysts were characterized using Fourier‐transform infrared spectroscopy (FT‐IR). As shown in Figure 5c, the significant absorption at 807 cm^−1^ is caused by the stretching vibration of the heptazine ring unit, while the broad band in the range of 1100–1700 cm^−1^ represents the stretching vibration mode of the aromatic C‐N heterocyclic skeleton in the typical CN structure. The weak absorption in the range of 3000–3500 cm^−1^ is owning to the stretching vibration of the N─H bond in the ─NH_x_ group and the O‐H stretching vibration from the physical adsorption of hydroxyl groups or H_2_O molecules.^[^ ^22^ ^]^ The peaks at 1606 and 1410 cm^−1^ in ZIS correspond to the adsorption peaks of water molecules and hydroxyl groups.^[^ ^23^ ^]^ In summary, it can be determined from XRD and FT‐IR that the composite material shows characteristic peaks of ZIS and CN, indicating the successful composite of the materials.

X‐ray photoelectron spectroscopy (XPS) is widely used to determine elemental composition and chemical states. As shown in Figure 5d, C, N, Zn, In, and S were determined in the S_0.5_‐CN/ZIS composite material using XPS, which is consistent with the EDS results. As shown in Figure 5e, the C 1s characteristic binding energy of CN is fitted with three peaks at 284.80 eV (surface amorphous carbon), 286.31 eV (C─NH_2_), and 288.23 eV (N─C═N).^[^ ^24^ ^]^ Since S replaced the N atoms in the lattice, the peak area of N─C═N significantly decreased. More importantly, the characteristic peaks of the composite material shift toward higher binding energies, while the shift of S‐CN/ZIS is more than that of CN/ZIS. The N 1s spectrum (Figure S8a, Supporting Information) exhibits a similar trend, while characteristic peaks of Zn 2p, In 3d, and S 2p (Figure S8b–d, Supporting Information) show the opposite trend. As is well known, an increase in the binding energy of an element signifies electron loss, while a decrease in binding energy indicates electron receiving. Therefore, the change in characteristic peaks indicates electron transfer between CN and ZIS, while S doping enhances this process.^[^ ^25^ ^]^ In addition, two peaks are observed in the S 2p spectrum of S_0.5_‐CN. The peak at 168.9 eV corresponds to the S─O bond, while the peak at 164.7 eV corresponds to the C─S─C bond, suggesting that S replaces the N atoms in the lattice (Figure 5f).^[^ ^26^ ^]^ The formation of flexible C─S─C bonds and appropriate temperature stimulation are the main reasons for the formation of the hollow coral‐like porous structure.

### 3.2 Visible Light Driven PHE

**Figure 6:**
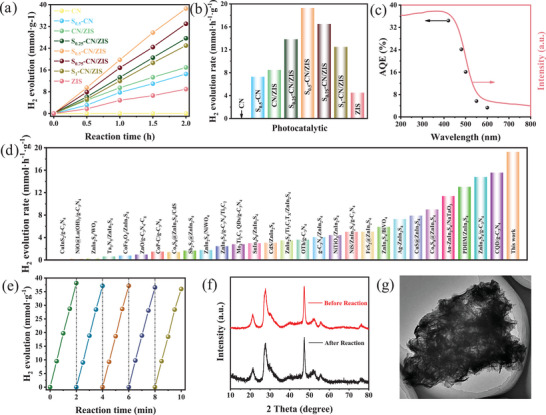
a) PHE performances with increasing time and b) PHE rates of different catalysts, c) wavelength‐dependent apparent quantum efficiency of S_0.5_‐CN/ZIS catalysts, d) comparison of PHE rates over S_0.5_‐CN/ZIS and other representatively reported catalysts, e) cyclic stability of S_0.5_‐CN/ZIS, f) XRD patterns of S_0.5_‐CN/ZIS before and after photocatalytic reaction, and g) TEM image of S_0.5_‐CN/ZIS catalyst after consecutive five cycles.

The PHE reaction with Na_2_SO_3_ (0.35 M) and Na_2_S·9H_2_O (0.25 M) as the hole scavenger under visible light irradiation (λ > 420 nm) was selected as a model process to evaluate S‐CN/ZIS heterojunction performance. Priorly, the optimal theoretical mass ratio of CN to ZIS in the CN/ZIS heterojunction was studied. When 0.05 g, 0.1 g, and 0.15 g of CN were added respectively, the H_2_ evolution rates of CN‐0.05/ZIS, CN‐0.1/ZIS, and CN‐0.15/ZIS are shown in Figure S9 (Supporting Information). The PHE rate for CN‐0.1/ZIS is 8.463 mmol g^−1^ h^−1^, which is higher than that of CN‐0.05/ZIS (7.385 mmol g^−1^ h^−1^) and CN‐0.15/ZIS (6.735 mmol g^−1^ h^−1^). As shown in **Figure** **6**a, CN and ZIS exhibit poor performance after a 2 h reaction at room temperature, while S‐CN has significantly better performance than CN. In comparison, S_0.5_‐CN/ZIS shows the highest hydrogen production activity. As shown in Figure 6b, CN and ZIS exhibit PHE rates for only 0.006 mmol g^−1^ h^−1^ and 4.472 mmol g^−1^ h^−1^, respectively. This low rate could result from rapid charge carrier recombination and insufficient active sites on the surface. Notably, the PHE rate of S_0.5_‐CN was significantly increased to 7.260 mmol g^−1^ h^−1^, which was better than that of CN and even CN loaded with Pt co‐catalyst (CN+Pt, 5.973 mmol g^−1^ h^−1^). This increase is attributed to the optimization of the morphological structure, band structure, and H adsorption–desorption process of S_0.5_‐CN by S doping. Moreover, the PHE rate of the CN/ZIS catalyst can reach up to 8.463 mmol g^−1^ h^−1^, indicating that the IEF enhances charge transfer, reduces carrier recombination, and thus improves PHE efficiency. The PHE rate of the optimized S_0.5_‐CN/ZIS catalyst is 19.252 mmol g^−1^ h^−1^, which is significantly higher than that of CN/ZIS. Besides, the hydrogen evolution performance of S_0.5_‐CN/ZIS was evaluated using different sacrificial agents such as Na_2_S/Na_2_SO_3_, EDTA, TEOA, TEA, lactic acid, ascorbic scid, MeOH, etc. As shown in Figure S10 (Supporting Information), due to the rapid hole consumption caused by the low oxidation potential of S^2−^ and SO_3_^2−^, Na_2_S/Na_2_SO_3_ system show the highest hydrogen evolution rate. It is worth emphasizing that the PHE efficiency of S_0.5_‐CN/ZIS is significantly higher than that of photocatalysts based on ZnIn_2_S_4_ and g‐C_3_N_4_ in most of recent reports (Figure 6d; Table S2, Supporting Information).

To confirm that H_2_ production is light‐driven, we measured the light absorption quantum efficiency (AQE) of S_0.5_‐CN/ZIS at various wavelength. As shown in Figure 6c, the AQE of S_0.5_‐CN/ZIS is closely related with its light absorption characteristics, indicating a positive correlation trend. At a wavelength of 420 nm, the S_0.5_‐CN/ZIS catalyst achieves an AQE of 34.43%, demonstrating the effective conversion of solar energy to hydrogen energy in the photocatalytic process. Besides, the S_0.5_‐CN/ZIS shows stable performance in the PHE reaction (Figure 6e). After being reused at least five times (10 h), its activity did not exhibit obvious decay, compared with the original performance. In the 5th run, the PHE efficiency of the S_0.5_‐CN/ZIS catalyst only decreased by about 5.7%, while high conversion efficiency maintained throughout the 5th reuse. Moreover, the microstructure and phase composition of the used catalyst showed minimal changes compared to the fresh S_0.5_‐CN/ZIS (Figure 6f,g).

As described above, S‐modified photocatalysts show excellent PHE performance. To reveal the reasons for the improved performance, their thermodynamic feasibility, band position, and light absorption characteristics were investigated. Figure S11a (Supporting Information) shows the UV–vis diffuse reflectance spectrum (UV–vis DRS). The pure CN has a weak response to visible light, with an absorption edge at around 460 nm. The absorption edge of S_0.5_‐CN shows a noticeable red shift and enhanced absorption intensity, indicating that S doping affects the intrinsic E_g_ of CN.^[^ ^26^ ^]^ The S_0.5_‐CN/ZIS has a stronger visible‐light absorption capacity compared to CN/ZIS. The E_g_ of the sample can be calculated using the Kubelka–Munk formula. The E_g_ of ZIS is 2.33 eV, while S_0.5_‐CN has a lower E_g_ of 2.62 eV compared to CN (2.80 eV). The decrease in E_g_ benefits light absorption and the generation of photo‐induced charge carriers, thereby improving the efficiency of PHE.

In addition, Mott–Schottky (M–S) measurement was conducted to determine the semiconductor type and the corresponding flat‐band potential (E_fb_). As shown in Figure S11b,c (Supporting Information), ZIS and CN exhibit positive slopes in the M–S plots, indicating the n‐type semiconductor characteristics. The E_fb_ of ZIS, CN, and S_0.5_‐CN are −0.33, −0.47, and −0.86 eV, respectively (relative to Ag/AgCl, pH = 7). When it is relative to NHE (E_NHE_ = E_Ag/AgCl_ + 0.197 eV), the E_fb_ are −0.13, −0.27, and −0.66 eV, respectively. In general, the E_fb_ of an n‐type semiconductor is approximately equal to the CB potential (E_CB_).^[^ ^27^ ^]^ Therefore, the valence band (VB) potentials (E_VB_) of ZIS, CN, and S_0.5_‐CN are 2.20, 2.47, and 1.96 eV, respectively, which are calculated by the equation E_g_ = E_VB_ – E_CB_. Based on the above analysis, the band structures of ZIS, CN, and S_0.5_‐CN are shown in Figure S11d (Supporting Information). Under visible light, the charge transfer path at the contacts between S_0.5_‐CN and ZIS can be only attributed to the S‐scheme mechanism or the traditional II‐type heterojunction.

**Figure 7:**
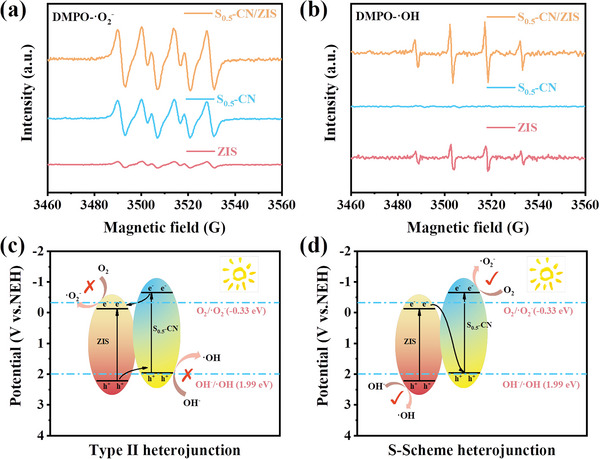
Schematic energy‐band diagram of CN, S_0.5_‐CN and ZIS, DMPO spin‐trapping EPR spectra of the samples in a) methanol for DMPO‐O_2_^−^ and b) water for DMPO‐OH under 420 nm light irradiation. c) Traditional II‐type and d) S‐scheme charge transfer mechanism for S_0.5_‐CN/ZIS heterojunction in DMPO trapping processes.

Electron paramagnetic resonance (EPR) spectroscopy is further used to study the specific charge transfer mechanisms.^[^ ^28^ ^]^ After visible light irradiation for 10 min, the DMPO‐O_2_^−^ signal is easy‐detectable on the S_0.5_‐CN and S_0.5_‐CN/ZIS samples, whereas the signal detected on ZIS is much weaker (**Figure** **7**a). Due to the E_CB_ (0.13 eV) in ZIS, it is thermodynamically difficult to reduce O_2_ to O_2_^−^. Because the S_0.5_‐CN/ZIS generates more photoelectrons, its signal strength is significantly stronger than that of S_0.5_‐CN. In contrast, samples containing ZIS show a strong DMPO‐OH signal, while S_0.5_‐CN could not exhibit an OH signal (Figure 7b). Based on the above evidence (especially the EPR signals of OH and O_2_^−^ radicals detected on S_0.5_‐CN/ZIS), the possibility of II‐type heterojunction charge transfer in S_0.5_‐CN/ZIS can be excluded (Figure 7c). Meanwhile, in situ XPS can more directly reflect the charge transfer pathways in heterojunction catalysts. As shown in Figure S12 (Supporting Information), the characteristic peaks of C and N in S_0.5_‐CN/ZIS shift to lower angles under light conditions, while Zn, In, and S shift to higher angles. This indicates that S_0.5_‐CN gains electrons, while ZIS loses electrons.^[^ ^29^ ^]^ As shown in Figure 7d, photogenerated electrons move from the CB of ZIS to the VB of S_0.5_‐CN at the S‐scheme heterojunction interface in the S_0.5_‐CN/ZIS composite. The transferred electrons from ZIS can effectively reduce recombination of intrinsic electrons and holes in the S_0.5_‐CN system, thereby increasing the surface charge density of the S_0.5_‐CN/ZIS photocatalyst.

### 3.3 Critical Role of S in Enhancing IEF and Optimizing Hydrogen Adsorption Energy

**Figure 8:**
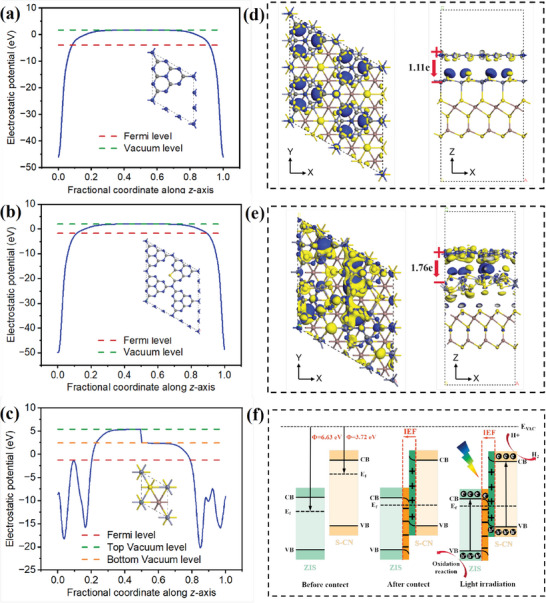
a–c) Work functions of CN, S‐CN_A_, and ZIS facets. 3D charge density difference at d) CN/ZIS and e) S‐CN_A_/ZIS interface. f) Schematic illustration of IEF‐induced S‐CN/ZIS S‐scheme heterojunction for H_2_O photo splitting under visible light irradiation. The blue and yellow zones in d,e) represent the charge accumulation and depletion, respectively.

In S_0.5_‐CN/ZIS heterogeneous catalysts, S doping plays a crucial role in adjusting the band structure, particularly the IEF. It is widely recognized that the interface between two semiconductors with varying Fermi levels generates an IEF, and the strength of the IEF increases with a greater disparity in Fermi levels.^[^ ^30^ ^]^ The work function (Φ) is a fundamental surface property of a material, which is calculated by the equation Φ = E_vac_ – E_f_, where E_vac_ is the vacuum energy level (assumed as 0 eV) and E_f_ is the Fermi level.^[^ ^31^ ^]^ As shown in **Figure** **8**, the Φ of ZIS, CN, and S‐CN_A_ (S replaces a nitrogen atom coordinated with two carbon atoms in the C_6_N_7_ unit) are 6.63, 5.69, and 3.72 eV, respectively. To verify the impact of different doping sites on the IEF, the Φ of S‐CN_C_ (S replaces a nitrogen atom coordinated with three carbon atoms in the C_6_N_7_ unit) and S‐CN_T_ (S replaces nitrogen atoms connected to three nearest pyridine rings) were also determined as 3.97 and 3.62 eV, respectively (Figure S13, Supporting Information). It can be concluded that S doping significantly decreases the work function of CN, resulting in a more noticeable difference in Fermi level between S‐CN and ZIS as well.

Furthermore, theoretical simulations were conducted to analyze the heterojunction interface between ZIS and CN, along with the 3D charge density variance, to determine the direction of IEF and the charge transfer mechanism. As shown in Figure 8d, electrons exhibit facile migration from the CN side to the ZIS side through the heterojunction interface. During this process, negative and positive charges are generated on sides near the interface of ZIS and CN, respectively. Therefore, a unique IEF is formed at the CN/ZIS interface with an electron transfer amount of 1.11 e. As shown in Figure 8e and S‐CN/ZIS interface exhibits a more stronger IEF effect (1.76 e). Moreover, doping S at different positions can also enhance the strength of IEF (Figure S10, Supporting Information). These data demonstrate that S doping has a significant enhancement effect on the IEF in S‐CN/ZIS system, regardless of the doping positions. And the direction of IEF is from S‐CN to ZIS.

Based on the above analysis, the PHE reaction mechanism for S‐CN/ZIS heterojunction could be proposed. As shown in Figure 8f, the photogenerated electrons spontaneously flow from the CB of ZIS to the VB of S‐CN due to the IEF effect in the S‐scheme heterojunction. And the electrons transported from the CB of ZIS can recombine with intrinsic holes in the VB of S‐CN. High‐energy electrons on the surface of S‐CN are involved in the reduction reactions with adsorbed H to produce H_2_. The charge transfer mode of the S‐scheme heterojunction promotes spatial separation of photo‐generated electrons and holes, providing the robust redox capability for efficient PHE reactions. Obviously, the unique charge transfer mode of S‐scheme heterojunction enables a large number of photogenerated electrons to accumulate on the surface of S‐CN, and S‐CN becomes the primary site for the PHE. Therefore, the advantage of S doping on CN can be fully utilized in the PHE process of S‐CN/ZIS heterojunction. In addition, S‐doping also further enhances the charge transfer of S‐scheme heterojunctions.

Due to the strong IEF effect of S‐modified heterojunctions on charge carrier separation and transfer in the PHE process, it is necessary to study the enhanced IEF effect on charge carrier behavior through photoelectrochemical properties. As shown in Figure S14a (Supporting Information), the photocurrent signal of pure CN exhibits the lowest value. Composite materials exhibit higher photocurrent signal levels, where the S_0.5_‐CN/ZIS catalyst displays the highest photocurrent response signal under visible light. This indicates that the separation of charge carriers is more effective, and charge transfer is significantly improved in S‐modified heterojunction materials. Photoluminescence (PL) spectra are frequently utilized to analyze surface structures and excited states. Electrons and holes in quasi‐equilibrium states recombine under photoexcitation to emit light, generating energy distributions at various wavelengths. As shown in Figure S14b (Supporting Information), the pure CN exhibits the highest PL intensity at an excitation wavelength of 380 nm, suggesting that a large number of photo‐generated charge carriers easily recombine within CN. The PL intensity of S_0.5_‐CN is significantly lower than that of CN, suggesting that S doping effectively enhances the separation and transfer of photo‐generated charge carriers. Note that the S_0.5_‐CN/ZIS heterojunction shows the lowest PL signal after S doping, suggesting that the enhanced IEF significantly improves charge separation and suppresses recombination of e^−^–h^+^ pairs effectively. It is well known that electrochemical impedance spectroscopy (EIS) techniques are commonly used to measure the charge transfer resistance, efficiency of separating photo‐induced e^−^–h^+^ pairs, and material conductivity. As shown in Figure S14c (Supporting Information), pure CN exhibits the largest semicircular Nyquist plot, suggesting the highest charge transfer resistance among all samples, while the EIS radius of the S_0.5_‐CN/ZIS photocatalyst is the smallest. This is attributed to the S doping increasing conductivity by enhancing the IEF as a critical factor for facilitating rapid charge transfer.^[^ ^32^ ^]^

**Figure 9:**
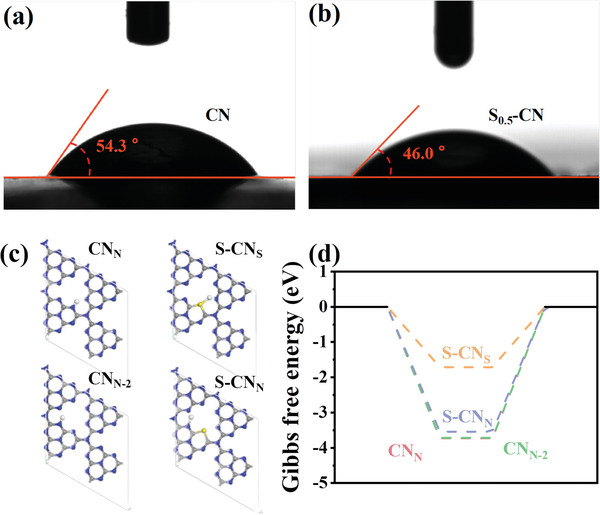
Water contact angle of a) CN and b) S_0.5_‐CN. c) Optimized structures of H* active intermediate for CN_N_, S‐CN_S_, CN_N‐2_, and S‐CN_N_. d) Hydrogen adsorption free energy of CN_N_, S‐CN_S_, CN_N‐2_, and S‐CN_N_ samples.

Obviously, the synergistic effect of S‐scheme heterojunction and S doping can induce more electrons to participate in the hydrogen production reaction on the S‐CN surface. The surface reactions for PHE include two main steps: the H adsorption at active sites to form active intermediates (H*) by combining electrons (H^+^+e^−^+*→H*), and the rapid H desorption to facilitate the release of H_2_ (2H*→H_2_+2*). According to the catalytic mechanism, the PHE reaction mainly takes place on the S‐CN surface. In order to investigate the effect of the hollow coral‐like porous structure on the adsorption and activation properties of water molecules, the water contact angles of CN and S‐CN were tested. As shown in **Figure** **9**a,b, CN has a contact angle of 54.3°, higher than that of S_0.5_‐CN (Figure 9b, 46.0°), suggesting that the latter has superior surface hydrophilicity. Therefore, the wrinkled surface, high porosity, and internal cavities allow S_0.5_‐CN to absorb more water molecules and release enough H for the PHE reaction.

It is well known that ΔG_H*_ is an accurate indicator for characterizing the intrinsic catalytic activity of PHE catalysts.^[^ ^33^ ^]^ For efficient PHE catalysts, the ideal ΔG_H*_ value should be close to 0 eV for the optimal H* adsorption and desorption processes. Therefore, the ΔG_H*_ values of N atoms (CN_N_) and S atoms (S‐CN_S_) at the same site before and after doping, as well as the ΔG_H*_ of N atoms around the doping site before and after doping (CN_N‐2_ and S‐CN_N_) were calculated to evaluate their intrinsic PHE catalytic activity. The optimization models of different adsorption structures are shown in Figure 9c. As shown in Figure 9d, the ΔG_H*_ value of S‐CN_S_ is closer to zero than that of CN_N_, suggesting that S could be a more efficient site for H adsorption and desorption. Note that due to the optimization in the adsorption–desorption process of H at N atoms near the S doping site, S‐CN_N_ has a closer ΔG_H*_ value to zero compared to CN_N‐2_. These indicate that the overall adsorption–desorption rate of H on the catalyst surface is enhanced, and thus adverse reactions resulted from electron accumulation were suppressed. In summary, S doping enhances the adsorption and activity of water molecules on the catalyst surface, as well as the adsorption and desorption of H, which is beneficial for PHE.

## 4 Conclusion

In conclusion, S‐CN/ZIS S‐Scheme heterojunction was successfully designed and prepared by in situ growth of ZIS on S‐CN framework. Under the appropriate temperature stimulation, the flexible C─S─C bonds allow the S‐CN/ZIS to form a special hollow coral‐like porous structure. The wrinkled surface, large specific surface area and porosity, and internal cavities facilitate the exposure of active sites, increased photo‐utilization, and adsorption and activation of water molecules in S‐CN/ZIS. The synergistic effect of S‐doping and S‐scheme heterojunction enhances the IEF, which promotes the separation and migration of photogenerated electron‐hole pairs, as well as the optimization for adsorption–desorption process of H, thus promoting the surface reduction reaction. These properties facilitate efficient water decomposition into H_2_ under visible light. The S_0.5_‐CN/ZIS catalyst shows superior PHE performance, with a hydrogen production rate of 19.252 mmol g^−1^ h^−1^ under visible light, which is 2.2 times higher than that of the CN/ZIS catalyst. Additionally, the AQE reaches 34.43% at 420 nm. The catalyst microstructure, enhanced IEF, adsorption and activation of H_2_O molecules, adsorption–desorption process of H on the surface, and S‐scheme heterojunction were investigated in detail by experimental and theoretical analyses, which demonstrated the important role of the synergistic effect of S doping and S‐scheme heterojunction in improving the hydrogen production efficiency. This study provides new ideas and theoretical basis for the design of CN‐based materials.

## Conflict of Interest

The authors declare no conflict of interest.

## Supporting information

Supporting Information

## Data Availability

The data that support the findings of this study are available from the corresponding author upon reasonable request.
