# Supplementary material for: In Situ Construction of Hollow Coral‐Like Porous S‐Doped g‐C3N4/ZnIn2S4 S‐Scheme Heterojunction for Efficient Photocatalytic Hydrogen Evolution
Source: Adv Sci (Weinh). 2024 Jul 3;11(33):2403771. doi: 10.1002/advs.202403771 (PMC11434114; doi:10.1002/advs.202403771)
Supplement: Supplementary file 1 — Supporting Information [file ADVS-11-2403771-s001.docx]

**Information**

**In situ construction of hollow coral-like porous S-doped g-C_3_N_4_/ZnIn_2_S_4_ S-scheme heterojunction for efficient photocatalytic hydrogen evolution**

Tianyu Wang ^1^, Xuanlin Pan ^1,2^, Minyi He ^1,2^, Lei Kang^1^,

Wangjing Ma ^1,^ *

^1^Technical Institute of Physics and Chemistry, Chinese Academy of Sciences, Zhongguancun East Road, Haidian District, Beijing, 100190

^2^University of Chinese Academy of Sciences, Zhongguancun East Road, Haidian District, Beijing, 100049

*E-mail: wjma@mail.ipc.ac.cn

**Text S1. Experimental reagents**

All chemicals used in this work are analytical grade chemicals. There was no purification step prior to the use of chemicals. Thiourea (CH_4_N_2_S, AR) and indium trichloride tetrahydrate (InCl_3_∙4H_2_O, AR) used in this work were from Aladdin Chemical Co., Ltd. Urea (CH_4_N_2_O, AR) was from Sinopharm Chemical Reagent Co., Ltd. Zinc chloride (ZnCl_2_, AR) from Shanghai Titan Technology Co., Ltd. Thioacetamide (TAA,AR), sodium sulfide hydrate (Na_2_S·9H_2_O, AR) and sodium sulfate (Na_2_SO_3_, AR) were from Meryer (Shanghai) Chemical Technology Co., Ltd. Deionized water was used for all operations of this work.

**Text S2. Characterization and analysis methods**

- 1. Characterization: The crystal phase and structure of the catalysts were obtained by XRD (Bruker D8 focus, Germany). The surface morphologies of the samples were determined by field emission SEM (FEI QUANTA FEG 250, USA) and the chemical composition of composites was studied through equipped EDS. The microstructure of the products was obtained by TEM (JEOL, JEM-2100F, Japan). BaSO_4_ was used as a reference, UV–vis DRS of the solid powders were recorded by an UV–vis spectrophotometer (Shimadzu, UV-2401 PC) equipped with an integrating sphere. The BET surface areas were estimated by nitrogen adsorption–desorption isotherms tested in nitrogen adsorption devices (Kantar Quadrasorb SI-MP, USA). The steady-state photoluminescence (PL) spectrum was detected with a spectrophotometer (Edinburgh FLS1000, UK) with an excitation wavelength of 314 nm. FTIR spectra were obtained from infrared spectrometer (Excalibur 3100, USA). The surface chemical state of solid powders was measured by XPS (ESCALAB 250Xi, UK). In-situ irradiated XPS measurements of samples with external light source irradiation were performed on the basis of XPS measurments. EPR spectra were performed on EPR spectrometer (Bruker E500, Germany). The generation of O_2_^-^ and OH was respectively detected in methanol and aqueous solution by using DMPO as trapping agent.
  2. Photocatalytic Measurements: Photocatalytic hydrogen evolution experiments were performed on a glass-enclosed gas circulation system (Labsolar-ⅢAG). Simulating sunlight using a 300 W Xe-lamp ( CEL-HXF300 ) coupled with a 420 nm cut-off filter. First, 50 mg sample was added into the 100 mL deionized water contained with 0.25 M Na_2_SO_3_·6H_2_O and 0.35 M Na_2_S·9H_2_O and then stirred for 30 min. Subsequently, vacuum pump was used to evacuate the dissolved oxygen from the solution. The reaction temperature was maintained at ca., 25 °C by circulating the cooling water during the photocatalytic reaction. The generated hydrogen was detected by an in-line gas chromatograph (GC-7900, Ar as carrier gas), collected once in the dark as a blank, and then injected once 30 min after irradiation. In the cyclic photocatalytic test, when one cycle test of 2 h was finished, the photocatalysts were collected by centrifugation before initiating the next cycle. The AQE was measured using different monochromatic light filters (420, 480, 500, 550, and 600 nm) after 1 h light irradiations, the test conditions were similar as the photocatalytic process. The AQE was calculated according to the following Equation 1:

where M is the amount (mol) of hydrogen in an hour, N_A_ is the Avogadro’s constant, c is the light velocity, h is the Planck constant, t is the photoreaction time, I is the light intensity, A is the irradiation area, and λ is the light wavelength.

- 1. Photoelectrochemical Measurements: Photoelectrochemical measurements were performed by a three-electrode system on CHI-660E (Chenhua CHI660E) electrochemical workstation. Pt electrode was used as the counter electrode, the reference electrode was saturated Ag/AgCl, the catalysts were spin-dropping on the FTO (fluorine-doped tin oxide (FTO) glass slide) as the working electrode, and the electrolyte was 0.5 M aqueous Na_2_SO_4_ solution. The working electrodes were prepared as following: Homogeneous catalyst ink was prepared by sonication of 2 mg/mL catalyst powder, deionized water, Nafion solution (5 wt%, Sigma-Aldrich) and absolute ethanol (the volume ratio was 8:0.5:1.5). Then, 20 μL slurry from above uniform mixture was drop-coated onto the FTO glass(1 cm × 1 cm) and then dried in air overnight.
  2. Theoretical Calculation Details: Density functional theory (DFT) calculations were performed in the Materials Studio software using the CASTEP module. The exchange-correlation interaction was defined by generalized gradient approximation (GGA) with the Perdew-Burke-Ernzerhof (PBE) functional. The energy cutoff and Monkhorst-Pack k-point mesh were set to 770 eV and 4 × 4 × 2, respectively. For the construction of surface models, a gap of 15 Å was used to eliminate interactions between periodic structures. The convergence criterions of energy and force calculations were set at 10^−5^ eV/atom and 0.05 eV/Å^−1^ , respectively. The relaxed lattice constant of AB stacking CN was a=b=7.10 Å, c=5.95 Å, while the lattice constant of ZIS bulk was a=b=3.91 Å, c=12.33 Å. The 2×2 slabs of monolayer S-CN and ZIS (001) were built to calculate their work functions, respectively. The calculation supercell of S-CN/ZIS (001) interface was selected as 2×2 unit cells of nomolayer S-CN siting on 4×4 ZIS (001). A vacuum gap more than 15 Å was adopted during the calculations. The work function (Φ) of a material can be calculated by the following equation: Φ = E_vac_ - E_F_, E_vac_ and E_F_ are the vacuum energy and fermi level energy, respectively.

The free energy calculation of hydrogen adsorption (ΔG_H*_) is based on Nørskovetals’ hydrogen electrode model, which was defined as follows:

ΔG_H∗_ = ΔE_H∗_ + ΔE_ZPE_ – TΔS

Where ΔE, ΔE_ZPE_, and ΔS respectively represent the changes of electronic energy, zero-point energy, and entropy that caused by adsorption of hydrogen. The ideal hydrogen adsorption free energy value (ΔG_H*_) is near zero, which could balance the adsorption and desorption of hydrogen reaction.

**Fig. S1** SEM of (a) CN, (b) S_0.5_-CN, (c) CN/ZIS and (d) S_0.5_-CN/ZIS.

**Fig. S2** SEM of S_0.5_-CN(450 °C).

**Fig. S3** TEM of S_0.5_-CN(550 °C).

**Fig. S4** SEM of ZIS.


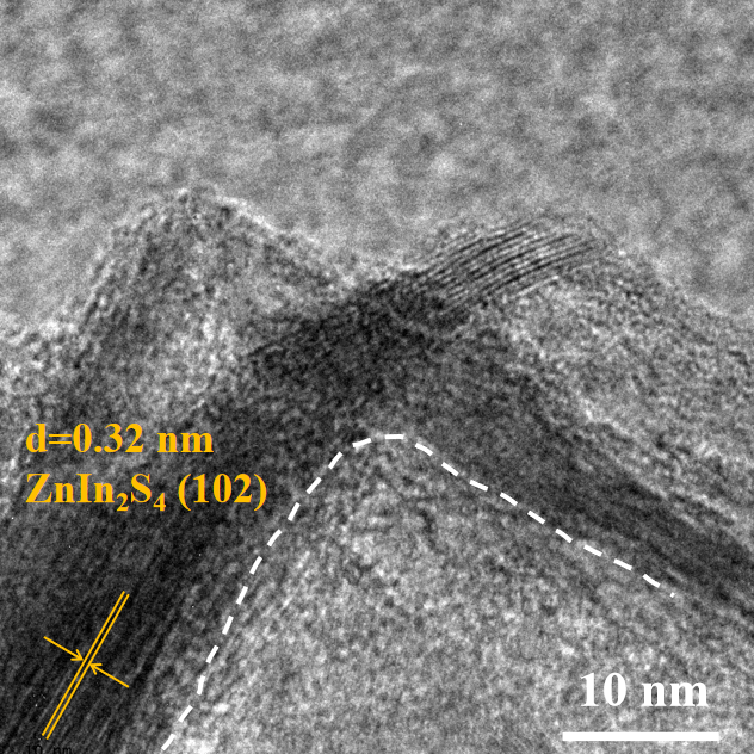


**Fig. S5** HRTEM of S_0.5_-CN/ZIS.

**Fig. S6** TEM-EDS line scan of S_0.5_-CN/ZIS (outer layer).

**Fig. S7** TEM-EDS line scan of S_0.5_-CN/ZIS (inner layer).

**Fig. S8** (a) N 1S high-resolution XPS spectra of CN, CN/ZIS and S_0.5_-CN/ZIS, (b) Zn 2p, (c) In 3d and (d) S 2p high-resolution XPS spectra of ZIS, CN/ZIS and S_0.5_-CN/ZIS.

**Fig. S9** Photocatalytic H_2_ production activity of different samples.

**Fig. S10** Photocatalytic H_2_ evolution rate of S_0.5_-CN/ZIS with different sacrificial agents, including Na_2_S/Na_2_SO_3_, EDTA, TEOA, TEA, lactic acid, ascorbic scid, MeOH.

**Fig S11.** (a) UV–vis DRS (band gaps), and (b), (c) Mott–Schottky plots of the photocatalysts, (d) Schematic energy-band diagram of CN, S_0.5_-CN and ZIS.

**Fig. S12** (a) High-resolution in-situ XPS spectra of C 1s (a), N 1s (b), Zn 2p (c), In 3d (d) and S 2p (e) of S_0.5_-CN/ZIS.

**Fig S13.** Work functions of (a) S-CN_T_ and (b) S-CN_C_, 3D charge density difference at (c) S-CN_T_/ZIS and (d) S-CN_C_/ZIS interface.

**Fig S14.** (a) Transient photocurrent responses, (b) PL plots and (c) EIS Nyquist plots of the photocatalysts.

**Table S1**. Specific surface area and pore size analysis data for CN, S_0.5_-CN, ZIS, CN/ZIS and S_0.5_-CN/ZIS.

| **Sample** | **S_BET_ (m^2^·g^-1^）** | **D_pore_ (nm)** | **V_pore_ (cm^3^·g^-1^)** |
| --- | --- | --- | --- |
| CN | 28.384 | 55.256 | 0.417 |
| S_0.5_-CN | 48.875 | 3.827 | 0.331 |
| ZIS | 111.421 | 12.191 | 0.337 |
| CN/ZIS | 89.691 | 13.259 | 0.269 |
| S_0.5_-CN/ZIS | 190.184 | 3.827 | 0.426 |

**Table S2.** Comparison of representative g-C_3_N_4_ and ZnIn_2_S_4_-based photocatalysts for H_2_ evolution efficiency.

| Photocatalysts | Light source | Sacrificial agent | Cocatalyst  /sensitizer | Activity  (mmol g ^-1^ h ^-1^) | AQE | Refs. |
| --- | --- | --- | --- | --- | --- | --- |
| CuInS_2_/g-C_3_N_4_ | 350 W Xe lamp  (λ>365 nm） | 10 vol% TEOA | / | 0.10 | / | S1^[1]^ |
| NiO@La(OH)_3_/g-C_3_N_4_ | 300 W Xe lamp  (λ>400 nm) | 10 vol% TEOA | / | 0.20 | / | S2^[2]^ |
| ZnIn_2_S_4_/WO_3_ | 300 W Xe lamp  (λ>400 nm) | 10 vol % methanol | / | 0.30 | 2.81%  (at 400 nm) | S3^[3]^ |
| Ta_3_N_5_/ZnIn_2_S_4_ | 300 W Xe lamp  (λ>400 nm) | 0.25 M Na_2_SO_3_ /0.35 M Na_2_S | / | 0.63 | 4.73%  (at 420 nm) | S4^[4]^ |
| CoFe_2_O_4_/ZnIn_2_S_4_ | 300 W Xe lamp  (λ>420 nm) | 20 vol% TEOA | 3 wt% Pt | 0.80 | 5.0%  (at 420 nm) | S5^[5]^ |
| ZnO/g-C_3_N_4_–C_g_ | 300 W Xe lamp  (λ>420 nm) | 10 vol% TEOA | 3 wt% Pt | 0.99 | 0.92%  (at 420nm) | S6^[6]^ |
| CoP–C/g-C_3_N_4_ | 300 W Xe lamp  (λ>400 nm) | 10 vol% TEOA | / | 1.50 | 3.03%  (at 400nm) | S7^[7]^ |
| Co_9_S_8_@ZnIn_2_S_4_/CdS | 300 W Xe lamp  (λ>420 nm) | 10% Na_2_SO_3_ | 3 wt% Pt | 1.42 | / | S8^[8]^ |
| Sb_2_S_3_/ZnIn_2_S_4_ | 250 W Xe lamp  (λ>420 nm) | 1 mmol TEOA | 3 wt% Pt | 1.69 | / | S9^[9]^ |
| ZnIn_2_S_4_/NiWO_4_ | 300 W Xe lamp  (λ>420 nm) | 10 vol% TEOA | / | 1.78 | / | S10^[10]^ |
| ZnIn_2_S_4_/g-C_3_N_4_/Ti_3_C_2_ | 300 W Xe lamp  (λ>420 nm) | 10 vol% TEOA | / | 2.45 | / | S11^[11]^ |
| Mo_2_Ti_2_C_3_ QDs/g-C_3_N_4_ | 300 W Xe lamp  (λ>420 nm) | 10 vol% TEOA | / | 2.81 | 3.80%  (at 420nm) | S12^[12]^ |
| SnIn_4_S_8_/ZnIn_2_S_4_ | 300 W Xe lamp  (λ>420 nm) | 10 vol% TEOA | 3 wt% Pt | 2.99 | / | S13^[13]^ |
| CdS/ZnIn_2_S_4_ | 300 W Xe lamp  (320-700 nm) | 0.25 M Na_2_SO_3_ /0.35 M Na_2_S | / | 3.07 | 15.90%  (at 420nm) | S14^[14]^ |
| ZnIn_2_S_4_/Ti_3_C_2_TX/ZnIn_2_S_4_ | 300 W Xe lamp  (λ>420 nm) | 10 vol% TEOA | 3 wt% Pt | 3.50 | 11.14%  (at 420nm) | S15^[15]^ |
| OTh/g-C_3_N_4_ | 300 W Xe lamp  (λ>420 nm) | 10 vol% ascorbic acid | 1 wt% Pt | 3.63 | 13.39%  (at 405nm) | S16^[16]^ |
| g-C_3_N_4_@ZnIn_2_S_4_ | 300 W Xe lamp  (λ>420 nm) | 25 vol% methanol | / | 4.08 | / | S17^[17]^ |
| NiTiO_3_/ZnIn_2_S_4_ | 3X 30W LEDs  (λ>420 nm) | 10 vol% TEOA | / | 4.43 | 4.39%  (at 450nm) | S18^[18]^ |
| NiS/ZnIn_2_S_4_/g-C_3_N_4_ | 300 W Xe lamp | 20 vol% TEOA | 1.5 wt% Pt | 5.02 | 30.5%  (at 420 nm) | S19^[19]^ |
| FeS_2_@ZnIn_2_S_4_ | 300 W Xe lamp  (AM 1.5 G) | 10 vol% TEOA | / | 5.05 | 13.4%  (at 420nm) | S20^[20]^ |
| ZnIn_2_S_4_/BiVO_4_ | 300 W Xe lamp  (λ>400 nm) | 10 vol% TEOA | 3 wt% Pt | 5.90 | / | S21^[21]^ |
| Ag-ZnIn_2_S_4_ | 300 W Xe lamp  (λ>420 nm) | 0.25 M Na_2_SO_3_ /0.35 M Na_2_S | / | 7.30 | 0.70%  (at 405nm) | S22^[22]^ |
| CuS@ZnIn_2_S_4_ | 300 W Xe lamp  (λ>400 nm) | 0.25 M Na_2_SO_3_ /0.35 M Na_2_S | / | 7.91 | 5.25%  (at 380nm) | S23^[23]^ |
| Co_9_S_8_/ZnIn_2_S_4_ | 300 W Xe lamp  (λ>400 nm) | 10 vol% TEOA | / | 9.04 | / | S24^[24]^ |
| Au-ZnIn_2_S_4_/NaTaO_3_ | 300 W Xe lamp  (Simulation of sunlight) | 10 vol% TEOA | / | 11.40 | 10.1%  (at 350nm) | S25^[25]^ |
| PDIIM/ZnIn_2_S_4_ | 300 W Xe lamp  (320-780 nm) | 0.25 M Na_2_SO_3_ /0.35 M Na_2_S | / | 13.04 | / | S26^[26]^ |
| ZnIn_2_S_4_/g-C_3_N_4_ | 300W Xe lamp  (λ>400 nm) | 0.25 M Na_2_SO_3_ /0.35 M Na_2_S | / | 14.80 | / | S27^[27]^ |
| CQD/g-C_3_N_4_ | 300 W Xe lamp  (λ>420 nm) | 10 vol% TEOA | 1 wt% Pt | 15.57 | / | S28^[28]^ |
| This work | 300 W Xe lamp  (λ>420 nm) | 0.25 M Na_2_SO_3_ /0.35 M Na_2_S | / | 19.25 | 34.43%  (at 420nm) | / |

**References**

[1] J. J. Zhang, Y. Zhao, K. Z. Qi, S. Y. Liu, *J. Mater. Sci. Technol.* **2024**, 172, 145.

[2] Y. H. Wang, L. Gao, J. Q. Huo, Y. W. Li, W. W. Kang, C. X. Zou, L. S. Jia, *Chem. Eng. J.* **2023**, 460, 11.

[3] M. Y. Zhao, S. Liu, D. M. Chen, S. S. Zhang, S. A. C. Carabineiro, K. L. Lv, *Chin. J. Catal.* **2022**, 43, 2615.

[4] X. Q. Zhan, Y. P. Zheng, B. Li, Z. Fang, H. L. Yang, H. T. Zhang, L. Y. Xu, G. Shao, H. L. Hou, W. Y. Yang, *Chem. Eng. J.* **2022**, 431, 12.

[5] C. X. Li, H. N. Che, P. W. Huo, Y. S. Yan, C. B. Liu, H. J. Dong, *J. Colloid Interface Sci.* **2021**, 581, 764.

[6] Z. Y. Li, Y. G. Chen, Y. H. Zhang, W. Ai, Q. Lei, T. J. Yao, D. Zhong, W. J. Liu, W. B. Jin, L. Yang, *J. Mater. Sci. Technol.* **2024**, 168, 35.

[7] Z. H. Huang, X. X. Long, M. Liu, X. P. Li, Y. X. Du, Q. Liu, Y. Chen, S. J. Guo, R. Z. Chen, *J. Colloid Interface Sci.* **2024**, 653, 1293.

[8] Y. Zhang, Y. X. Wu, L. Wan, H. J. Ding, H. X. Li, X. Y. Wang, W. H. Zhang, *Appl. Catal. B-Environ.* **2022**, 311, 11.

[9] Y. Xiao, H. Wang, Y. H. Jiang, W. L. Zhang, J. M. Zhang, X. Y. Wu, Z. C. Liu, W. Deng, *J. Colloid Interface Sci.* **2022**, 623, 109.

[10] M. Y. Zhang, P. F. Tan, L. Yang, H. H. Zhai, H. L. Liu, J. Y. Chen, R. F. Ren, X. Y. Tan, J. Pan, *J. Colloid Interface Sci.* **2023**, 634, 817.

[11] L. L. Wang, T. Yang, L. J. Peng, Q. Q. Zhang, X. L. She, H. Tang, Q. Q. Liu, *Chin. J. Catal.* **2022**, 43, 2720.

[12] L. Ding, Y. Y. Tang, S. Y. Wang, Y. Q. Zhang, X. Y. Chen, H. J. Zhou, *J. Colloid Interface Sci.* **2024**, 653, 1671.

[13] R. Z. Xiong, C. C. Tang, S. F. Liu, Y. H. Xiao, B. C. Cheng, S. J. Lei, *Sep. Purif. Technol.* **2022**, 295, 14.

[14] Y. W. Zhu, J. Chen, L. H. Shao, X. N. Xia, Y. T. Liu, L. L. Wang, *Appl. Catal. B-Environ.* **2020**, 268, 10.

[15] G. C. Zuo, Y. T. Wang, W. L. Teo, A. M. Xie, Y. Guo, Y. X. Dai, W. Q. Zhou, D. Jana, Q. M. Xian, W. Dong, Y. L. Zhao, *Angew. Chem.-Int. Edit.* **2020**, 59, 11287.

[16] Y. Li, X. L. Pang, Q. Zhao, B. M. Zhang, X. Guo, Y. Zhang, Y. Xie, C. L. Qin, L. Q. Jing, *ACS Appl. Mater. Interfaces* **2023**, DOI: 10.1021/acsami.2c2184913.

[17] Y. W. Xiao, B. Yao, M. H. Cao, Y. D. Wang, *Small* **2023**, 19, 13.

[18] S. Dhingra, M. Sharma, V. Krishnan, C. M. Nagaraja, *J. Colloid Interface Sci.* **2022**, 615, 346.

[19] X. Y. Ji, R. T. Guo, J. Y. Tang, Z. D. Lin, Y. Yuan, L. F. Hong, W. G. Pan, *J. Colloid Interface Sci.* **2022**, 618, 300.

[20] K. Y. Chen, Y. X. Shi, P. Shu, Z. Y. Luo, W. L. Shi, F. Guo, *Chem. Eng. J.* **2023**, 454, 11.

[21] J. D. Hu, C. Chen, Y. Zheng, G. P. Zhang, C. X. Guo, C. M. Li, *Small* **2020**, 16, 10.

[22] R. R. Pan, M. Hu, J. Liu, D. F. Li, X. D. Wan, H. Z. Wang, Y. M. Li, X. M. Zhang, X. L. Wang, J. Jiang, J. T. Zhang, *Nano Lett.* **2021**, 21, 6228.

[23] H. T. Fan, Z. Wu, K. C. Liu, W. S. Liu, *Chem. Eng. J.* **2022**, 433, 10.

[24] G. P. Zhang, D. Y. Chen, N. J. Li, Q. F. Xu, H. Li, J. H. He, J. M. Lu, *Angew. Chem.-Int. Edit.* **2020**, 59, 8255.

[25] J. H. Zhang, H. J. Gu, X. L. Wang, H. H. Zhang, S. Y. Chang, Q. Li, W. L. Dai, *J. Colloid Interface Sci.* **2022**, 625, 785.

[26] L. Liu, Y. Wu, R. Song, Y. Zhang, Y. F. Ma, J. Wan, M. L. Zhang, H. L. Cui, H. Yang, X. L. Chen, J. J. Wang, *J. Colloid Interface Sci.* **2022**, 628, 701.

[27] M. X. Tan, Y. Ma, C. Y. Yu, Q. J. Luan, J. J. Li, C. B. Liu, W. J. Dong, Y. J. Su, L. J. Qiao, L. Gao, Q. P. Lu, Y. Bai, *Adv. Funct. Mater.* **2022**, 32, 8.

[28] L. Zhou, H. Z. Guo, Z. M. Wang, L. Wang, *Carbon* **2023**, 213, 9.
